# Pathophysiologic Mechanisms of Cardiovascular Disease in Obstructive Sleep Apnea Syndrome

**DOI:** 10.1155/2013/521087

**Published:** 2013-06-27

**Authors:** Carlos Zamarrón, Luis Valdés Cuadrado, Rodolfo Álvarez-Sala

**Affiliations:** ^1^Division of Respiratory, Hospital Clínico Universitario, c/Travesia de la Choupana s/n, A Coruña, 15706 Santiago, Spain; ^2^Division of Respiratory, Hospital Universitario La Paz, Paseo de la Castellana 261, 28046 Madrid, Spain

## Abstract

Obstructive sleep apnea syndrome (OSAS) is a highly prevalent sleep disorder, characterized by repeated disruptions of breathing during sleep. This disease has many potential consequences including excessive daytime sleepiness, neurocognitive deterioration, endocrinologic and metabolic effects, and decreased quality of life. Patients with OSAS experience repetitive episodes of hypoxia and reoxygenation during transient cessation of breathing that provoke systemic effects. Furthermore, there may be increased levels of biomarkers linked to endocrine-metabolic and cardiovascular alterations. Epidemiological studies have identified OSAS as an independent comorbid factor in cardiovascular and cerebrovascular diseases, and physiopathological links may exist with onset and progression of heart failure. In addition, OSAS is associated with other disorders and comorbidities which worsen cardiovascular consequences, such as obesity, diabetes, and metabolic syndrome. Metabolic syndrome is an emerging public health problem that represents a constellation of cardiovascular risk factors. Both OSAS and metabolic syndrome may exert negative synergistic effects on the cardiovascular system through multiple mechanisms (e.g., hypoxemia, sleep disruption, activation of the sympathetic nervous system, and inflammatory activation). It has been found that CPAP therapy for OSAS provides an objective improvement in symptoms and cardiac function, decreases cardiovascular risk, improves insulin sensitivity, and normalises biomarkers. OSAS contributes to the pathogenesis of cardiovascular disease independently and by interaction with comorbidities. The present review focuses on indirect and direct evidence regarding mechanisms implicated in cardiovascular disease among OSAS patients.

## 1. Introduction 

Obstructive sleep apnea syndrome (OSAS) is a common disorder characterized by recurrent upper airway collapse during sleep [1]. This results in a reduction or complete cessation of airflow despite ongoing inspiratory efforts and leads to arousals, sleep fragmentation, and oxyhemoglobin desaturation [2].

A spectrum of sleep related obstructed breathing has been described in the literature [3]. This ranges from snoring [4], and upper airway resistance syndrome [5] to obesity hypoventilation syndrome [6]. The focus of the current review is OSAS, which lies in between these two extremes.

Though clinically recognized since the 1960s [7], general awareness of OSAS has been slow to develop. OSAS has been associated with cardiovascular disease [8], automobile accidents [9], chronic obstructive pulmonary disease (COPD) [10], heart failure [11] and health related quality of life deterioration [12].

Another emerging public health problem is metabolic syndrome, which represents a constellation of cardiovascular risk factors. OSAS often coexists with obesity and has been shown to be independently associated with insulin resistance, which is an important component of metabolic syndrome [1, 13]. Given the current obesity epidemic, the prevalence of both metabolic syndrome and OSAS is on the rise.

The present review analyzes the relation between OSAS and cardiovascular disease and how it may be affected by OSAS-associated disorders and comorbidities. 

## 2. OSAS Epidemiology

A variety of epidemiological studies have demonstrated the high prevalence of OSAS and its relation to cardiovascular risk factors (Table 1). Durán et al. in 2001, performed 555 complete polysomnographies and found sleep disordered breathing, defined as AHI > 5, in 26.3% of men and 28% of women. AHI was associated with hypertension after adjusting for age, sex, BMI, neck circumference, alcohol use, and smoking habit [14]. In India, Udwadia et al. found a 19.5% prevalence of sleep disordered breathing, defined as AHI > 5, and 7.5% prevalence of OSAS, defined as AHI > 5 with symptoms. BMI, neck circumference and diabetes mellitus were found to be associated with sleep disordered breathing [15]. Sharma et al. reported a 13.7% overall prevalence of sleep disordered breathing and 3.6% prevalence of OSAS. Multivariate analysis revealed that male gender, age, obesity, and waist/hip ratio were significant risk factors for OSAS [16].

Pływaczewski et al. found a 7.5% prevalence of OSAS. OSAS was found to be an independent predictor of coronary artery disease after adjusting for age, sex, BMI, neck circumference, and smoking habit [17].

As age advances, sleep breathing related difficulties become increasingly common. Several OSAS studies in older populations report little or no association of OSAS with sleepiness, hypertension, or decrements in cognitive function [18, 19]. 

## 3. OSAS and Cardiovascular Diseases Mechanisms

**Figure 1:**
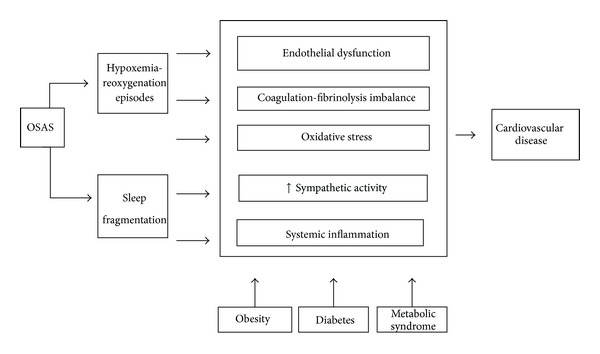
A schematic summary of the proposed sequence of events in obstructive sleep apnea syndrome starting from episodic hypoxia and sleep fragmentation.

 The mechanisms involved in the association between OSAS and vascular diseases are complex and diverse. Patients with OSAS experience repetitive episodes of hypoxia and reoxygenation during transient cessation of breathing that may provoke systemic effects. These patients also present increased levels of biomarkers linked to endocrine-metabolic and cardiovascular alterations. The relation between OSAS and cardiovascular disease involves a number of mechanisms such as the following (Figure 1).

### 3.1. Sleep Fragmentation

The importance of sleep to health and cardiovascular disease has become increasingly apparent. Percentage time in slow wave sleep has been inversely associated with incident hypertension (regardless of sleep duration and fragmentation) and sleep-disordered breathing. In fact, selective deprivation of slow wave sleep may contribute to adverse blood pressure in older men [20]. Bekci et al. found that total antioxidant capacities were decreased in the higher arousal index, suggesting that patients with higher arousal index may be more prone to vascular events [21].

In OSAS, severe sleep fragmentation disturbs nocturnal renin and aldosterone secretion profiles and increases nighttime urine excretion. CPAP treatment has been reported to improve sleep, restore plasma renin activity and aldosterone oscillations, and lower nocturnal urine natriuresis and diuresis [22]. Møller et al. found that long-term CPAP (Continuous positive airway pressure) reduced blood pressure, which was correlated with reductions in plasma renin and angiotensin II levels [23].

Extreme sleep habits can affect health and have been associated with increased inflammation. Significant changes in habitual sleep duration can lead to chronic low-grade systemic inflammation [24] and activation of proinflammatory pathways may represent a mechanism. In a study involving pediatric OSAS patients, increased TNF-*α* levels were primarily driven by sleep fragmentation and BMI. These levels were closely associated with the degree of sleepiness. Surgical treatment of OSAS resulted in significant reductions in TNF-*α* levels and reduction in sleepiness [25]. 

### 3.2. Enhanced Sympathetic Traffic

In OSAS, there is enhanced sympathetic traffic through a tonic activation of chemoreflex activity that normalizes with CPAP treatment [26, 27]. OSAS-associated disturbances, especially chronic intermittent hypoxia and enhanced sympathetic activity, lead to upregulation of the renin-angiotensin system and downregulation of nitric oxide synthases [28]. When an obstructive apnea occurs, it is terminated by a sudden arousal, that is, lightening of sleep or awakening from sleep [29]. The cyclic intermittent hypoxia provides the causal link between upper airway obstruction during sleep and sympathetic activation during awakening. Cyclic intermittent hypoxia may lead to sympathoexcitation via two mechanisms: first, augmentation of peripheral chemoreflex sensitivity (hypoxic acclimatization) and, second, direct effects on sites of central sympathetic regulation. 

In a study in healthy humans, intermittent hypoxia significantly increased sympathetic activity and daytime blood pressure after a single night of exposure. The baroreflex control of sympathetic outflow declined [30]. Surges in sympathetic nervous system activity associated with apneic events have also been related to antifibrinolytic activity reflected by elevations in PAI-1 [31]. 

Increased sympathetic activity and intermittent hypoxia associated with apneic episodes has been proposed as a possible mechanism behind the association between OSAS, systemic inflammation and cardiovascular disease. CPAP reduces sympathetic nerve activity [32], increases arterial baroreflex sensitivity [33], and decreases vascular risk [34]. 

### 3.3. Oxidative Stress

In OSAS patients, increased production of superoxide by neutrophils [35], increased biomarkers of lipid peroxidation [36], and increased levels of 8-isoprostanes [37] have been observed. There is an emerging consensus that OSAS is an oxidative stress disorder. 

Apnea produces a decline in oxygen levels followed by reoxygenation when breathing resumes. Cyclical episodes of hypoxia-reoxygenation, which are analogous to cardiac ischemia/reoxygenation injury, may cause ATP depletion and xanthine oxidase activation and increases the generation of oxygen-derived free radicals. CPAP therapy decreases the levels of oxidative stress in OSAS patients [38, 39].

In a study involving children with OSAS, Malakasioti et al. found increased hydrogen peroxide levels in exhaled breath condensate, which is an indirect index of altered redox status in the respiratory tract [40].

Oxidative stress can profoundly regulate the cellular transcriptome through activation of transcription factors, including specificity protein-1, hypoxia-inducible factor-1, c-jun, and possibly NF*κβ*. Activation of redox-sensitive gene expression is suggested by the increase in some protein products of these genes, including vascular endothelial growth factor [41], erythropoietin [42], and endothelin-1 [43]. Low oxygen tension is a trigger for activation of polymorphonuclear neutrophils, which adhere to the endothelium [44]. 

Increased oxidative stress has been associated with development of cardiovascular diseases and can be promoted by the chronic intermittent hypoxia characteristic of OSAS [45]. A variety of studies suggest that oxidative stress is present in OSAS at levels relevant to tissues such as the arterial wall [46, 47]. This process enhances lipid uptake into human macrophages and may contribute to atherosclerosis in OSAS patients [48]. Furthermore, OSAS decreases blood antioxidant status in high-BMI subjects and may change the relationship between oxidative stress markers [49]. After CPAP, expression of eNOS and phosphorylated eNOS was found to be significantly increased, whereas expression of nitrotyrosine and nuclear factor-kappaB was significantly decreased [50]. However, other studies have shown that CPAP may not affect antioxidant defense [51]. Nair reported that oxidative stress is mediated, at least in part, by excessive NADPH oxidase activity. This author suggests that pharmacological agents targeting NADPH oxidase may provide a therapeutic strategy in OSAS [52].

### 3.4. Systemic Inflammation

In OSAS, intense local and systemic inflammations are present. Insofar as local inflammation, bronchial and nasal changes are especially relevant [53]. In a study by Carpagnano et al., OSAS patients showed a significant increase in IL-8 and ICAM concentrations in both plasma and exhaled condensate. In addition, they showed a higher neutrophil percentage in induced sputum. These findings were significantly and positively correlated to AHI [54]. In a recent study of 80 nonsmoking males, Cofta et al., found a progressive increase in the concentrations of three selectins with the severity of OSAS [55].

Adhesion of circulating leukocytes to the endothelial cells is considered one of the initial steps in the pathogenesis of atherosclerosis. The repetitive hypoxia-reoxygenation episodes associated with apneas and hypopneas in OSAS upregulate the production of inflammatory mediators and the expression of adhesion molecules. Different studies have reported changes in circulating levels of adhesion molecules in OSAS patients [56, 57]. Dyugovskaya et al. analysed polymorphonuclear apoptosis and expression of adhesion molecules in vitro in patients with moderate to severe OSAS. Decreased apoptosis and increased expression of adhesion molecules were observed. Although adhesion molecules may facilitate increased polymorphonuclear-endothelium interactions, decreased apoptosis may further augment these interactions and facilitate free radical and proteolytic enzymes [58].

OSAS patients present increased levels of inflammatory mediators such as TNF*α* and IL-6 [59, 60] that decrease with CPAP treatment [61, 62]. 

Systemic inflammation is increasingly being recognized as a risk factor for a number of complications including atherosclerosis [63] and is a well-established factor in the pathogenesis of cardiovascular disease [64]. Serum amyloid A is a major acute-phase protein in humans that has been associated with cardiovascular disease [65]. Levels of this protein are chronically elevated in patients with OSAS [66] and improve with CPAP [67]. 

C-reactive protein is an important serum marker of inflammation with major implications for cardiovascular morbidity and atherogenesis [68]. C-reactive protein levels are increased in OSAS in accordance with disease severity [69–71] and are decreased after CPAP treatment [72, 73].

The mechanisms by which inflammation contributes to OSAS-induced vascular dysfunction are not known. Reoxygenation after a brief period of hypoxia as experienced repetitively and systematically by OSAS patients may predispose to cell stress, possibly because of mitochondrial dysfunction. It has been suggested that such events favor the activation of a proinflammatory response as mediated through the transcription factor nuclear NF*κβ*, a master regulator of inflammatory gene expression. The downstream effects of this activation include increased expression of inflammatory cytokines which may contribute to endothelial dysfunction and subsequently cardiovascular complications [74]. 

 Inflammation may be an important link between increased sympathetic nervous system activity and vascular dysfunction in OSAS. Chronically elevated sympathetic activity produced an inflammatory response in several organs and vascular beds [75].

Some authors point to the role of the T lymphocyte. This cell is known to play an important role in angiotensin II-induced hypertension and endothelial dysfunction via NADPH oxidase-induced superoxide production [76].

Increased expression of inflammatory cytokines may contribute to endothelial dysfunction and subsequent cardiovascular complications. Currently, some studies suggest that pentraxin 3, an acute phase response protein, is rapidly produced and released by several cell types, especially mononuclear phagocytes and endothelial cells in response to primary inflammatory signals. Pentraxin 3 may play a significant role in OSAS-associated vascular damage [77]. Arnaud et al. reported that some inhibition of molecules such as RANTES/CCL5, a cytokine that selectively attracts memory T lymphocytes and monocytes, may play a significant role in athesrosclerosis remodeling and OSAS-associated vascular damage [78]. 

However, mesenchymal stem cells triggered an early anti-inflammatory response in rats subjected to recurrent obstructive apneas, suggesting that these stem cells could play a role in the physiological response to counterbalance inflammation in OSAS [79].

In healthy human males, Querido et al. analysed the effect over 10 days of nightly intermittent hypoxia in the following systemic inflammatory markers: serum granulocyte macrophage colony-stimulating factor, interferon-*γ*, interleukin 1*β*, interleukin 6, interleukin 8, leptin, monocyte chemotactic protein-1, vascular endothelial growth factor, intracellular adhesion molecule-1, and vascular cell adhesion molecule-1. There was no significant change in any of the markers. These findings suggest that a more substantial or a different pattern of hypoxemia might be necessary to activate systemic inflammation, that the system may need to be primed before hypoxic exposure, or that increases in inflammatory markers in OSAS patients may be more related to other factors such as obesity or nocturnal arousal [80]. 

### 3.5. Hypercoagulability

Hypercoagulability resulting from increased coagulation or inhibited fibrinolysis is associated with an increased risk for cardiovascular disease [81, 82]. This is another factor implicated in its association with OSAS [83, 84].

A variety of findings support the existence of a relation between hypercoagulability, OSAS, and cardiovascular disease. Firstly, patients with OSAS present higher plasma levels of several procoagulant factors such as fibrinogen [85], activated clotting factor FVII (FVIIa), FXIIa, and thrombin/antithrombin III complexes [86], platelet activity [87], and the fibrinolysis-inhibiting enzyme plasminogen activator inhibitor (PAI-1) [88, 89]. Secondly, increased D-dimer levels in untreated OSAS have been correlated with severity of nocturnal hypoxemia, characteristic of OSAS [90]. Von Känel et al., found that OSAS patients showed lower mesor (mean) and amplitude (difference between maximum and minimum activity) of D-dimer. However, there were no significant differences in changes of periodic pattern and in day/night rhythm parameters of prothrombotic markers pre- to posttreatment between the CPAP and placebo condition [91].

Thirdly, sleep fragmentation and sleep efficiency data have been associated with increased levels of von Willebrand factor and soluble tissue factor, two markers of a prothrombotic state [92]. 

### 3.6. Endothelial Dysfunction

Endothelial dysfunction is an early marker of vascular abnormality preceding clinically overt cardiovascular disease [93–95]. It is known from years ago that endothelial dysfunction identified in the peripheral vasculature strongly predicts coronary disease [96].

The intact endothelium regulates vascular tone and repair capacity, maintaining proinflammatory, anti-inflammatory, and coagulation homeostasis. Alteration of these homeostatic pathways results in endothelial dysfunction before structural changes in the vasculature. The hypoxia, hypercapnia, and pressor surges accompanying obstructive apneic events may serve as potent stimuli for the release of vasoactive substances. Levels of nitric oxide, a major vasodilator substance released by the endothelium, have been found to be decreased in OSAS patients, and these levels normalize with CPAP therapy [97]. 

In OSAS, endothelial dysfunction could be caused by both hypoxia-reoxygenation cycles and chronic sleep fragmentation produced by repetitive arousals. A causal relationship between OSAS and endothelial dysfunction was demonstrated by a study in which flow-mediated dilation in the forearm was improved by CPAP treatment [98, 99]. Levels of nitric oxide, a major vasodilator substance released by the endothelium, have been found to be decreased in OSAS patients, and these levels normalize with CPAP therapy [100]. 

A number of studies involving OSAS patients indicate an associated endothelial dysfunction [101–103] that improves after CPAP [104, 105]. In addition to the fact that OSAS comorbidities (e.g., hypertension, diabetes, hyperlipidaemia, and smoking) may result in endothelial dysfunction, OSAS itself may be an independent risk factor. 

Among the most important vasoconstrictive substances is endothelin-1, a peptide hormone secreted under the influence of hypoxia [106]. Several studies have reported higher endothelin-1 levels in OSAS patients [107, 108]; however, Grimpen et al. report conflicting findings [109]. This divergence might be explained by differences in study design. The groups studied by Phillips and Saarelainen had more severe disease and, thus, underwent more severe oxygen desaturations that acted as a trigger for endothelin-1 secretion. Gjørup et al. showed that hypertensive OSAS patients had greater nocturnal and diurnal endothelin-1 plasma levels than healthy controls, suggesting that OSAS does not affect plasma endothelin-1 levels in the absence of coexistent cardiovascular diseases [110].

The inconsistency of the above endothelin-1 levels likely reflects the predominantly abluminal release of endothelin. Using rat models of arterial hypertension, several authors have reported elevated vascular production of endothelin-1, while circulating levels remained similar to controls [111, 112]. This demonstrates that circulating levels of endothelin-1 do not exclude elevated vascular production in OSAS. 

In recent years, endothelial progenitor cells have gained a central role in vascular regeneration and endothelial repair capacity through angiogenesis and restoring endothelial function of injured blood vessels. Endothelial dysfunction is frequently present in OSAS [113] and may have a potential role in the pathogenesis of vascular diseases that is pertinent to OSAS [114].

Furthermore, It has been reported that microvascular endothelial function is affected by OSAS predominantly through increased oxidative stress, and treatment of OSAS may improve endothelial function mainly by reducing oxidative stress [115, 116].

### 3.7. Vibration Resulting from Snoring

Snoring associated vibration energy transmission from the upper airway to the carotid artery has been hypothesized as a potential atherosclerotic plaque initiating and rupturing event that may provide a pathogenic mechanism linking snoring and embolic stroke. The vibration produced by snoring could lead to vessel wall damage in the carotid arteries [117, 118]. 

In animals models, Howitt et al. demonstrated the transmission of oscillatory pressure waves from the upper airway lumen to the peripharyngeal tissues and across the carotid artery wall to the lumen [119]. Cho et al. found carotid arteries subjected to continuous pericarotid tissue vibration displayed endothelial dysfunction, suggesting a direct plausible mechanism linking heavy snoring to the development of carotid atherosclerosis [120]. Although intriguing, this concept requires further study.

## 4. Obesity

OSAS often coexists with obesity and many epidemiological studies have demonstrated the existence of an association. Significant OSAS is present in approximately 40% of obese individuals, and about 70% of OSAS patients are obese [121]. Young et al. estimated that the majority of severe OSAS cases (58%) were due to obesity [122]. In fact, obesity parameters such as BMI, neck circumference, and visceral fat accumulation have been identified as the most important predictors of OSAS [123, 124]. 

Obesity is one of the major cardiovascular risk factors associated with OSAS. The OSAS-obesity association may have an influence on other disorders, such as cardiovascular diseases. Vgontzas et al. found a strong independent association between OSAS, visceral obesity, and insulin resistance. This author demonstrated that male obese patients with OSAS had a greater amount of computerised tomography-determined visceral adipose tissue in the abdomen than a group of BMI-matched men without OSAS [125]. Moreover, increased abdominal fat accumulation has been singled out as an independent risk factor for cardiovascular diseases [126]. It has been suggested that upper abdominal obesity is more insulin resistant and releases metabolically active products into the portal circulation. 

The mechanism by which obesity can favor the onset of OSAS is not well known, but it could be that central obesity precipitates or exacerbates OSAS because fat deposits in the upper airway affect distensibility [127]. The increased volume of abdominal fat could predispose to hypoventilation during sleep and/or reduce the oxygen reserve, favoring oxygen desaturation during sleep [128]. In recent years, much attention has been focused on the interaction between OSAS and products released by adipose tissue such as leptin, adiponectin, resistin, and ghrelin [129].

### 4.1. Leptin

Leptin is an adipocyte-derived hormone that regulates body weight through control of appetite and energy expenditure [130]. Furthermore, leptin is a cytokine and is therefore also involved in the inflammatory process. Several studies have shown increased levels of leptin in OSAS suggesting its role in the disease [131–133]. The mechanisms underlying the relation between leptin and OSAS are very diverse and may involve overnight changes in apnea levels [134, 135], sleep hypoxemia [136], and hypercapnia [137]. 

A direct relationship between OSAS and leptin is supported by the fact that effective OSAS treatment with CPAP also influences leptin levels [138, 139]. Although the precise mechanism explaining the effect of CPAP has not yet been elucidated, it can be inferred that reduction in sympathetic activity [140] and improvement in insulin sensitivity play a role [141]. 

Leptin levels have been proposed as a prognostic marker for OSAS [142, 143] and have been implicated in the pathogenesis of OSAS and related cardiovascular disease [132, 144, 145]. Leptin's role had been recently extended into that of participant to oxidative stress, although its exact role in this process is yet to be defined. Elevated leptin levels correlate significantly with several indices of OSAS disease severity such as nocturnal hypoxemia. Leptin may be a counteractive mechanism against chronic intermittent hypoxia-related oxidative stress and may also be a marker for atherosclerosis risk [146].

### 4.2. Adiponectin

Adiponectin is an adipocyte-derived cytokine with regulatory functions in glucose and lipid metabolism. It also has profound anti-inflammatory and antiatherogenic effects. Levels of plasma adiponectin are decreased in obesity and metabolic syndrome [147, 148]. OSAS has independently been associated with reduced levels of adiponectin [149, 150] which may favour cardiovascular disease development. The recurrent hypoxia-reoxygenation attacks in OSAS patients may activate oxidative stress and lead to low levels of adiponectin [151]. 

 Some authors have observed that serum adiponectin levels may be independent of the degree of OSAS [132]. Decreased adiponectin may result from increased sympathetic activity [152] and higher levels of cytokines such as IL-6 and TNF*α* [153]. In fact, there are conflicting reports as to whether CPAP treatment of OSAS effectively normalizes adiponectin levels [154, 155].

Obesity has been implicated in the relation between OSAS and adiponectin [156]. In a study involving media under hypoxic conditions in an ex vivo mouse model, adiponectin secretion was measured. In obese mice, hypoxic stress reduced adiponectin in the supernatant of mesenteric fat tissue but not subcutaneous fat tissue. These findings suggest that abdominal obesity, representing abundant mesenteric fat tissue susceptible to hypoxic stress, partly explains adiponectin levels in OSAS patients, and that reduction of visceral fat accumulation may combat OSAS-related atherosclerotic cardiovascular diseases in abdominal obesity [157].

### 4.3. Resistin

Resistin is a white adipose tissue hormone whose function has yet to be established. Evidence suggests that resistin is involved in pathological processes leading to cardiovascular disease including inflammation, endothelial dysfunction, thrombosis, angiogenesis, and smooth muscle cell dysfunction [158]. In a study of 20 obese OSAS patients, Harsch et al. found that CPAP treatment of OSAS had no significant influence on resistin levels [159]. In OSAS patients, hypoxic stress during sleep may enhance resistin production, possibly mediating systemic inflammatory processes. Through its effect on OSAS, CPAP therapy may help control resistin production [160].

### 4.4. Ghrelin

Ghrelin is a hormone that influences appetite and fat accumulation and its physiological effects are opposite to those of leptin. Current experimental evidence suggests that ghrelin may act centrally to decrease sympathetic nervous system activity through peripheral afferent nerve [161]. Thus, administration of ghrelin might become a unique new therapy for cardiovascular diseases [162].

In a study of 30 obese OSAS patients, Harsch et al. found that plasma ghrelin levels were significantly higher in OSAS patients than in controls. These elevated ghrelin levels could not be explained by obesity alone, since they rapidly decreased with CPAP therapy [163]. In a study of 55 consecutive OSAS patients, the study group presented significantly higher serum ghrelin levels than controls. No significant difference was noted in the levels of leptin, adiponectin, and resistin. There was a significant positive correlation between ghrelin and AHI [164]. 

Increased ghrelin levels have been found to support the presence of increased appetite and caloric intake in obese patients with OSAS, which in turn may further promote the severity of the underlying conditions [165]. In obese children, OSAS is associated with daytime sleepiness, elevation of proinflammatory cytokines, increased leptin, and decreased adiponectin [166]. However, in a recent study, OSAS patients with excessive daytime sleepiness were associated with increased circulating hypocretin-1 and decreased circulating ghrelin levels. This relationship is independent of AHI and obesity [167].

## 5. OSAS and Insulin Resistance

Dysglycemia and diabetes also increase the risk of developing cardiovascular disease [168]. With respect to OSAS, Mondini and Guilleminault found increased frequency of abnormal breathing during sleep in both lean and obese diabetics [169]. Elmasry et al. studied 116 hypertensive men and found a 36% prevalence of severe OSAS in diabetes patients compared to 15% in controls [170]. West et al. involving men with type 2 diabetes also reported a very high prevalence of OSAS (23%) [171, 172]. Several studies have reported that diabetic subjects with autonomic neuropathy, regardless of severity, had a relatively high prevalence of OSAS (26% and 30%). [153, 173]. 

OSAS might be a manifestation of an endocrine/metabolic abnormality with a strong role played by insulin resistance [174–176]. A variety of studies based on animal models have shown that hypoxia can alter glucose homeostasis [177, 178]. Polotsky et al. described that long-term exposure to intermittent hypoxia increased levels of insulin and glucose intolerance in obese, leptin-deficient mice [179]. Humans exposed to hypoxia present worsened glucose tolerance [180]. 

Most studies involving OSAS and insulin resistance have demonstrated an association between these two diseases, regardless of obesity [181, 182]. In a large population-based study involving normoglycemic hypertensive men, Resnick et al. found that the severity of OSAS was associated with increased insulin resistance [183]. Insulin resistance is associated with states of inflammation. Monocyte chemoattractant protein-1 levels are elevated in OSAS and may be involved in the pathogenesis of insulin resistance in these patients [184, 185]. 

## 6. Metabolic Syndrome and OSAS 

Metabolic syndrome is an emerging public health problem that represents a constellation of cardiovascular risk factors [186]. The association of metabolic syndrome with cardiovascular disease was already observed more than 40 years ago [187]. In addition, Reaven confirmed that metabolic syndrome was a well-established risk factor for cardiovascular disease [188]. 

The diagnosis of metabolic syndrome is based on a variety of criteria. According to the National Cholesterol Education Program (NCEP) guidelines, a diagnosis of metabolic syndrome requires three or more of the following risk factors: waist circumference 102 cm, triglycerides  1.7 mmol/L, HDL cholesterol < 1.04 mmol/L, blood pressure  130/85 mmHg, and fasting glucose 6.1 mmol/L [189]. 

**Figure 2:**
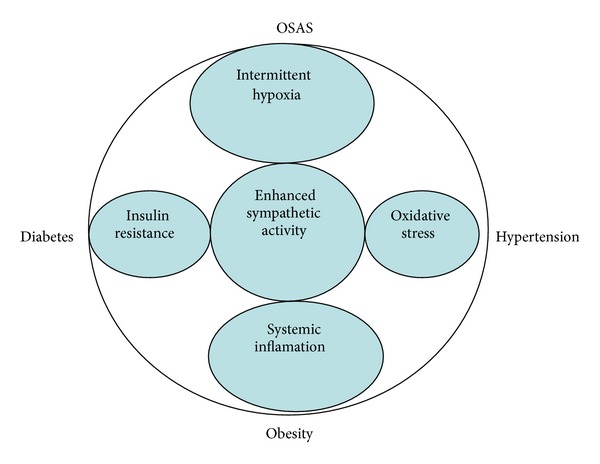
Obstructive sleep apnea syndrome and metabolic syndrome. Current perspective.

The prevalence of metabolic syndrome is markedly higher among OSAS patients. Ambrosetti et al. studied 89 consecutive OSAS patients and found metabolic syndrome in 53% of them [190]. Obese OSAS patients may have an increased rate of metabolic syndrome and higher levels of serum lipids, fasting glucose, leptin, and fibrinogen than obese subjects without OSAS. Thus, clinicians should be encouraged to systematically evaluate the presence of metabolic abnormalities in OSAS and vice versa [191] (Figure 2).

A number of previous epidemiological studies have found links between OSAS and metabolic syndrome. Vgontzas et al. reported that fasting glucose and insulin levels were significantly higher in OSAS patients compared to weight-matched control subjects. They also found that OSAS led to systemic inflammation and metabolic syndrome [192]. Gruber et al. prospectively studied 38 subjects with OSAS and 41 controls. After adjusting for age, BMI, and smoking, OSAS patients were found to be nearly six times more likely to have metabolic syndrome than control group [193]. In a 255-subject study by Lam et al. a similar likelihood was reported [194]. Shina et al. reported that C-reactive protein was higher in patients with both OSAS and metabolic syndrome [195]. Bonsignore et al. conclude that the metabolic syndrome occurs in about half of OSAS patients, irrespective of daytime sleepiness, and is a reliable marker of insulin resistance [196].

Both OSAS and metabolic syndrome may exert negative synergistic effects on the cardiovascular system through multiple mechanisms [197, 198]. In a study by Su et al., metabolic factors such as a higher BMI and fasting blood glucose and a lower HDL-cholesterol level were more strongly associated with elevated cardiovascular disease than with OSAS severity, suggesting that metabolic parameters are important contributors to cardiovascular diseases and should be corrected in patients with OSAS [199]. In a double-blind, placebo-controlled trial in OSAS patients, 3 months of CPAP therapy lowers blood pressure and partially reverses metabolic abnormalities [200]. 

## 7. OSAS and COPD

COPD is a systemic disease with multiple effects on end-organs including organs in the cardiovascular system [201]. Patients with diagnosed and treated COPD are at increased risk for hospitalizations and deaths due to cardiovascular diseases [202, 203].  Several studies have focused on the relation between endothelial dysfunction and COPD [204–206]. 

Systemic inflammation is the main atherothrombotic abnormality in COPD, but hypoxia-related platelet activation, procoagulant status, and oxidative stress may play a role [207, 208]. Mills et al. showed that patients with COPD have increased arterial stiffness and blood pressure in comparison with controls matched for age and smoking status [209]. Furthermore, there is evidence that COPD patients have a perturbed neurohumoral regulatory system leading to sympathovagal imbalance [210, 211]. This process may be related to chronic respiratory or metabolic conditions that manifest hypoxia, hypercapnia, and acidosis and elicit a maladaptive autonomic and inflammatory response [212].

OSAS may coexist with COPD and this combination has been the focus of extensive study. Flenley referred to it as “overlap syndrome” [213]. Overlap patients present more nocturnal desaturation than patients with either OSAS or COPD alone [214]. Individuals with overlap syndrome are at greater risk for pulmonary hypertension, right heart failure, and hypercapnia than patients who have either COPD or OSAS alone [215]. 

As inflammatory diseases, both OSAS and COPD are associated with higher cardiovascular risk. The mechanisms that may be involved include vascular inflammation, endothelial dysfunction, and tonic elevation of sympathetic neural activity. In sum, OSAS is one of the most frequent COPD comorbidities and may bring on increased inflammation [216–218]. The overlap syndrome is associated with an increased risk of death and hospitalization because of COPD exacerbation. CPAP treatment was associated with improved survival and decreased hospitalizations in patients with overlap syndrome [219]. Treatment consists of CPAP or noninvasive positive pressure ventilation, with or without associated O2, for correction of the upper airway obstructive episodes and hypoxemia during sleep [220, 221].

## 8. Conclusions

OSAS and intermittent hypoxia are associated with early vascular changes. Animal and clinical data support a specific role for intermittent hypoxia in promoting cellular changes at the vascular wall level thus triggering atherosclerosis. Independently, OSAS impairs endothelial function by altering regulation of endothelial vasomotor tone and repair capacity while promoting vascular inflammation and oxidative stress. 

There is increasing evidence of a causal relationship between OSAS and metabolic dysfunction. OSAS, by intermittent hypoxia, may induce or exacerbate various aspects of metabolic syndrome. Clinical studies show that OSAS can affect glucose metabolism, cholesterol, and inflammatory markers. Identification of OSAS as a potential causative factor in metabolic syndrome would have significant clinical impact and could improve the management and understanding of both disorders.

The association of OSAS with endocrine-metabolic and cardiovascular alterations indicates that, more than a local abnormality, OSAS should be considered a systemic disease. A vicious cycle may also appear involving hypoxemia-reoxygenation cycles, oxidative stress, and elaboration of proinflammatory cytokines promoting a more generalized inflammatory state. 

Sleep apnea research is an intriguing field providing considerable contributions to the cardiovascular literature with exciting insights for clinicians, basic scientists, and epidemiologists. 

## Figures and Tables

**Table 1:** Prevalence studies on obstructive sleep apnea syndrome.

Study	Population	Age	Method	Criteria	SDB prevalence	OSAS
Durán et al. 2001 [14]	2148 subjects from the electoral census	30–70	(1) Questionnaire (2) Validated portable instrument in 442 subjects (3) PSG in 555 subjects	AHI ≥ 5	26.3% (M) and 28% (F)	
				AHI ≥ 10	19% (M) and 14.9% (F)	
				AHI ≥ 15	14.2% (M) and 8.6% (F)	
				AHI ≥ 20	9.6% (M) and 6% (F)	
				AHI ≥ 30	6.8% (M) and 4.3% (F)	
				OSAS = AHI ≥ 10 plus Symptoms		3.4% M 3% F

Udwadia et al., 2004 [15]	658 healthy urban Indian subjects	35–65	(1) Questionnaire (2) PSG on subgroup in 250 subjects	AHI ≥ 5	19.5%	7.5%
				AHI ≥ 10	11.1%	6.1%
				AHI ≥ 15	8.4%	5.4%
				OSAS = AHI plus Symptoms		

Sharma et al., 2006 [16]	2150 semiurban community in Delhi	30–60	(1) Questionnaire (2) PSG on subgroup in 150 subjects	AHI ≥ 5	13.7%	
				OSAS = AHI ≥ 5 plus Symptoms		3.6%

Pływaczewski et al., 2008 [17]	1503 from Warsaw electoral registers	Over 30 years of age	(1) Questionnaire (2) PSG on subgroup in 676 subjects	AHI ≥ 10	14.3%	
				OSAS = AHI ≥ 10 plus Symptoms		7.5%

Abbreviations: AHI: apnea hypopnea index; PSG: polysomnography; SDB: sleep disordered breathing; M: male; F: female.

## Abbreviations

OSAS: Obstructive sleep apnea syndrome
CPAP: Continuous positive airway pressure
COPD: Chronic obstructive pulmonary disease
AHI: Apnea hypopnea index
BMI: Body mass index
PAI: Plasminogen activator inhibitor
ATP: Adenosine triphosphate
eNOS: Endothelial nitric oxide synthase
NF*κβ*: Nuclear factor-kappaB
NADPH: Nicotinamide adenine dinucleotide phosphate
IL-8: Interleukin 8
IL-6: Interleukin 6
ICAM: Intercellular adhesion molecule-1
RANTES: Regulated and normal T cell expressed and secreted
CCL5: Chemokine (C-C motif) ligand 5
TNF*α*: Tumor necrosis factor alpha
HDL: High-density lipoprotein.
